# Understanding the Molecular Determinants Driving the Immunological Specificity of the Protective Pilus 2a Backbone Protein of Group B *Streptococcus*

**DOI:** 10.1371/journal.pcbi.1003115

**Published:** 2013-06-27

**Authors:** Annalisa Nuccitelli, C. Daniela Rinaudo, Barbara Brogioni, Roberta Cozzi, Mario Ferrer-Navarro, Daniel Yero, John L. Telford, Guido Grandi, Xavier Daura, Martin Zacharias, Domenico Maione

**Affiliations:** 1 Novartis Vaccines and Diagnostics, Siena, Italy; 2 Institute of Biotechnology and Biomedicine (IBB), Universitat Autònoma de Barcelona (UAB), Bellaterra, Spain; 3 Catalan Institution for Research and Advanced Studies (ICREA), Barcelona (UAB), Spain; 4 Physics Department, Technical University Munich, Munich, Germany; University of California San Diego, United States of America

## Abstract

The pilus 2a backbone protein (BP-2a) is one of the most structurally and functionally characterized components of a potential vaccine formulation against Group B *Streptococcus*. It is characterized by six main immunologically distinct allelic variants, each inducing variant-specific protection. To investigate the molecular determinants driving the variant immunogenic specificity of BP-2a, in terms of single residue contributions, we generated six monoclonal antibodies against a specific protein variant based on their capability to recognize the polymerized pili structure on the bacterial surface. Three mAbs were also able to induce complement-dependent opsonophagocytosis killing of live GBS and target the same linear epitope present in the structurally defined and immunodominant domain D3 of the protein. Molecular docking between the modelled scFv antibody sequences and the BP-2a crystal structure revealed the potential role at the binding interface of some non-conserved antigen residues. Mutagenesis analysis confirmed the necessity of a perfect balance between charges, size and polarity at the binding interface to obtain specific binding of mAbs to the protein antigen for a neutralizing response.

## Author Summary

Group B *Streptococcus* (GBS) is the leading cause of neonatal invasive diseases and pili, as long filamentous fibers protruding from the bacterial surface, have been discovered as important virulence factors and potential vaccine candidates. The bacterial surface is the main interface between host and pathogen, and the ability of the host to identify molecular determinants that are unique to pathogens has a crucial role for microbial clearance. Here, we describe a strategy to investigate the immunological and structural proprieties of a protective pilus protein, by elucidating the molecular mechanisms, in terms of single residue contributions, by which functional epitopes guide bacterial clearance. We generated neutralizing monoclonal antibodies raised against the protein and identified the epitope region in the antigen. Then, we performed computational docking analysis of the antibodies in complex with the target antigen and identified specific residues on the target protein that mediate hydrophobic interactions at the binding interface. Our results suggest that a perfect balance of shape and charges at the binding interface in antibody/antigen interactions is crucial for the antibody/antigen complex in driving a successful neutralizing response. Knowing the native molecular architecture of protective determinants might be useful to selectively engineer the antigens for effective vaccine formulations.

## Introduction

The bacterial surface is the foremost interface between host and pathogen, and recognition of the specific epitopes by the immune system provides the host a key signature to initiate microbial clearance. Identification and characterization of antigenic epitopes is a rapidly expanding field of research with potential contributions to the tailored design of improved, safe and effective vaccines [1], [2]. A number of approaches are currently being used that require atomic-level information in understanding the rules governing antibody/antigen interaction, in particular it is the degree of complementarity between surfaces on epitope and paratope that determines the affinity and specificity of this interaction [3]–[5]. To date, the concept of complementariness is directly related to the conservation of the amino acid sequence on a specific neutralizing epitope. A single amino acid change resulted crucial to alter the surface antigenic properties of a specific epitope of Neuraminidase (NA) in Influenza virus [6].

*Streptococcus agalactiae* (also known as Group B *Streptococcus* or GBS) is a Gram-positive pathogen causing severe diseases in newborn and young infants worldwide [7]. Pilin proteins, structural components of cell surface-exposed appendages, have been discovered in GBS as important virulence factors as well as promising vaccine candidates [8]. These high molecular weight structures are made by a major shaft subunit (named backbone protein, BP), a major ancillary protein (named AP1), and a minor ancillary protein (named AP2). BP is distributed regularly along the pilus structure and is fundamental for pilus assembly whereas the two ancillary proteins are dispensable [9]. AP1 is thought to be located at the tip of the assembled pilus structure, while AP2 is involved in pilus attachment to the cell wall [10]–[12]. In GBS three pathogenicity islands, named Pilus Island-1 (PI-1), Pilus Island-2a (PI-2a) and Pilus Island-2b (PI-2b), each encoding pilin protective subunits, have been identified [9]. Among them, the backbone protein of Pilus Island 2a (BP-2a) is a key component of a promising pilus-based vaccine formulation against Group B *Streptococcus* infections [13], [14]. However, this protein showed the highest level of gene variability among all pilin antigens, characterized by six non cross-protective allelic variants [13]. Each variant identified was able to induce protective immunity in mouse models and opsonophagocytosis killing of live bacteria, but only against GBS strains expressing the homologous variant [13], [14]. A recent Structural Vaccinology approach applied to the BP-2a protein led to the identification of the minimal protein domain carrying the protective epitopes [14]. By using *in vitro* opsonophagocytosis assays and *in vivo* animal infectious models, we demonstrated that, within each variant, the domain D3 is responsible for eliciting neutralizing antibodies against pathogen homologous infections [14]. This structure-based approach combined with immunological assays succeeded in the generation of an easy-to-produce chimeric antigen capable to elicit protection against the majority of circulating GBS serotypes [14]. However, the specific mechanisms by which antibodies raised against each variant can mediate a neutralizing response only against GBS strains expressing the homologous variant are not completely understood.

The aim of this work is to contribute a deeper understanding of the molecular basis driving the immunogenic specificity of single BP-2a variants, explaining the mechanism by which amino acid variability on the antigen surface may allow the bacteria to adapt to the host environment and/or escape its immune system. To investigate the variant-specific immunogenicity of BP-2a at the molecular level, we generated neutralizing monoclonal antibodies (mAbs) raised against a specific allelic variant of the protein, the 515 allele [13], [14]. The produced mAbs were functionally screened according to their ability to recognize the polymerized pilus structure on the bacterial surface and to mediate opsonophagocytosis GBS killing *in vitro*. By Surface Plasmon Resonance (SPR) technology we determined their binding affinity. An approach based on partial digestion, immunocapture and mass spectrometry was used to identify the epitope region in the antigen. Finally, docking and molecular simulation prediction studies were used to elucidate the intrinsic and functional affinity between mAbs and antigen at a residue level.

This work describes a potential strategy to investigate the immunological and structural properties of a surface virulence factor, by elucidating at the molecular level the chemical-physical properties directly related to an effective neutralizing response against pathogen infection.

## Results

### Generation of mouse monoclonal antibodies targeting surface-exposed epitopes of the pilin protein BP-2a 515 variant

**Figure 1:**
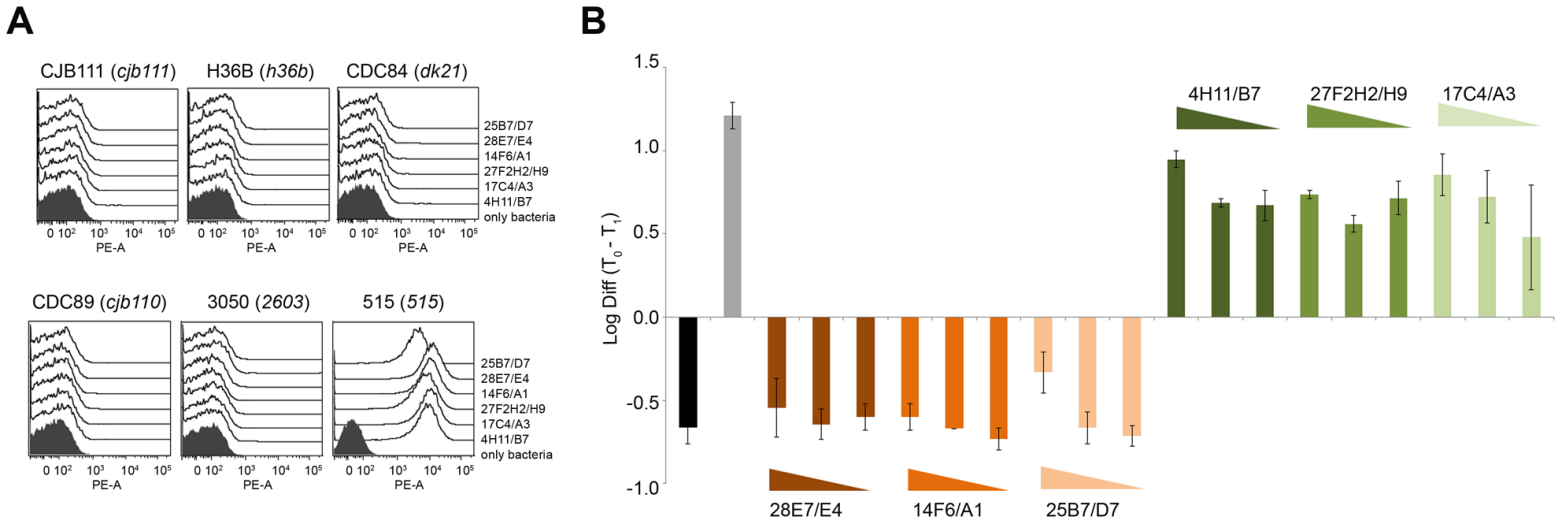
Selected mAbs against BP-2a 515 variant recognize only the polymerized pilus structures of the homologous strain and can mediate complement-dependent bacterial clearance. (A) Flow cytometry analysis on whole GBS strains stained with mAbs raised against BP-2a 515 variant. Six GBS strains expressing different BP-2a variants were used in the assay, strain CJB111 (cjb111 allele); strain H36B (h36b allele); strain CDC84 (dk21 allele); strain CDC89 (cjb110 allele); strain 3050 (2603 allele) and strain 515 (515 allele). Fixed bacteria were stained with monoclonal antibodies and then labeled with R-Phycoerythrin conjugated goat anti-mouse secondary antibodies. Black filled histograms indicate staining of bacteria with only secondary antibody. (B) Opsonophagocytosis activity of the selected six monoclonal antibodies. 10^4^ CFUs of GBS strain 515 were incubated for 1 h with differentiated HL60 cells, baby rabbit complement and monoclonal antibody (1∶30, 1∶90, and 1∶270 dilutions). The log_10_ differences between GBS colony-forming units at time 0 (10^4^ CFU) and time 1 h are shown. The mAbs used are recorded above each bar. Black shaded bars represent negative control (without baby rabbit complement); grey bars correspond to the polyclonal serum specific for the full length BP-2a 515 protein, used as positive control. Error bars indicate standard deviation from two independent experiments.

Recent advances in monoclonal antibodies (mAbs) technology suggested us to use them as tool to investigate the principles governing functional antibody/antigen interactions [6]. So, to understand the immunological differences among the six different variants of the highly immunogenic GBS protein BP-2a through the identification of neutralizing epitope(s), mouse monoclonal antibodies (mAbs) against the 515 allelic variant were generated following standard procedures (see Materials and Methods). Since surface accessibility of bacterial proteins is a fundamental pre-requisite of antibodies for mediating an effective humoral response against bacterial infections, selection criteria for mAb identification were based on variant-specificity and on bacterial surface staining, which was investigated by Flow Cytometry (FACS) analysis. The screening procedure resulted in the identification of six different monoclonal antibodies (named 4H11/B7, 17C4/A3, 27F2H2/H9, 14F6/A1, 25B7/D7, 28E7/E4) able to recognize only the polymeric pilus structure on the bacterial surface of their homologous strain 515 (**Figure 1A**). In fact, they were not able to stain the surface of GBS strains expressing a different BP-2a variant (**Figure 1A**).

To assess if the six selected mAbs could also mediate a functional immunogenic response against GBS we performed an *in vitro* opsonophagocytosis assay, using as effector cells differentiated HL60 cells, as described in the Materials and Methods section, and GBS strain 515. We analyzed each monoclonal antibody at three different dilutions in presence of baby rabbit complement. As shown in **Figure 1B**, only three out of six mAbs (4H11/B7, 17C4/A3 and 27F2H2/H9) were able to mediate an effective complement-dependent opsonization and killing of GBS bacteria, meaning that these mAbs can recognize and bind neutralizing epitopes exposed in the pilin protein on the bacterial surface.

### Biochemical characterization of selected mAbs

Classes and subclasses of the six monoclonal antibodies were determined as described in the Materials and Methods section. Clone 4H11/B7 secreted the IgG2b subclass; clone 17C4/A3 and 27F2H2/H9 secreted the IgG2a subclass whereas clones 14F6/A1, 25B7/D7 and 28E7/E4 had the IgG1 isotype.

To evaluate the interaction between BP-2a 515 variant and the selected mAbs, we conducted SPR (Surface Plasmon Resonance) analyses. A convenient strategy to study this interaction is to capture the antibody on a surface containing Fc-receptors in order to place the antibody in a well-defined orientation for binding analysis. Two CM5 biosensors, one coated with Protein A and the second with Protein G, were prepared in order to steadily capture the different isotypes of the mAbs and study their interaction with BP-2a 515 variant in terms of association (k_a_) and dissociation (k_d_) rate constants, and binding affinity (K_D_ = k_d_/k_a_).

The monoclonal antibodies 17C4/A3 and 27F2/H2/H9 were captured on both Protein A and Protein G biosensors while 4H11/B7 mAb was stably captured only in presence of Protein G. Two out of the three IgG1 mAbs, 25B7/D7 and 28E7/E4, were captured by Protein G, increasing the RU (refractive unit) of capture according to the concentration (5 or 15 nM), while 14F6/A1 was not captured by protein G up to the concentration of 15 nM. After the capture, the mAbs (4H11/B7, 17C4/A3 and 27F2H2/H9) that were the same antibodies that were able to mediate opsonophagocytic killing of GBS cells could bind BP-2a 515 variant, while the two IgG1 mAbs captured by the Protein G biosensor did not bind to the protein in the range of 0.5 to 2.5 µM. For the mAbs which were able to bind the BP-2a 515 variant single cycle kinetics were performed on both biosensors when possible. The average of three independent runs is reported in **Table 1**. Data showed that the association phase (k_a_) resulted comparable for all the tested mAbs, within a range of ∼2.5-fold (k_a_ max/k_a_ min). Larger differences were measured in the kinetic of dissociation kinetics (k_d_), with the most stable binding observed for 4H11/B7, k_d_ approximately 10-fold slower than for 17C4/A3 and 5.5-fold slower than for 27F2/H2/H9 mAb. Nevertheless, the corresponding thermodynamic dissociation constants (K_D_) did not differ among the three mAbs, which showed to strongly bind the BP-2a 515 variant.

**Table 1:** Kinetic analyses of 17C4/A3, 27F2/H2/H9 and 4H11/B7 mAbs interacting with BP-2a 515 variant.

mAb	k_a_ (1/Ms)	k_d_ (1/s)	K_D_ (nM)
17C4/A3	9.6±3.3 E+04	9.2±3.3 E-04	9.6±1.1
27F2H2/H9	8.6±3.0 E+04	4.7±1.3 E-04	5.7±1.3
4H11/B7	3.9±0.2 E+04	8.5±0.7 E-05	2.2±0.1

The association (k_a_) and dissociation (k_d_) rate constants, and the binding affinity (K_D_) are the average of three independent runs for each monoclonal antibody. For mAb 25B7/D7, 28E7/E4 and 14F6/A1 (15 nM), it was not possible to determine the kinetic parameters since the first two did not binding to GBS 59 (0.5–2.5 µM) while the third could not be captured on Protein G biosensor.

### Mapping with functional mAbs identifies an epitope in domain D3

**Figure 2:**
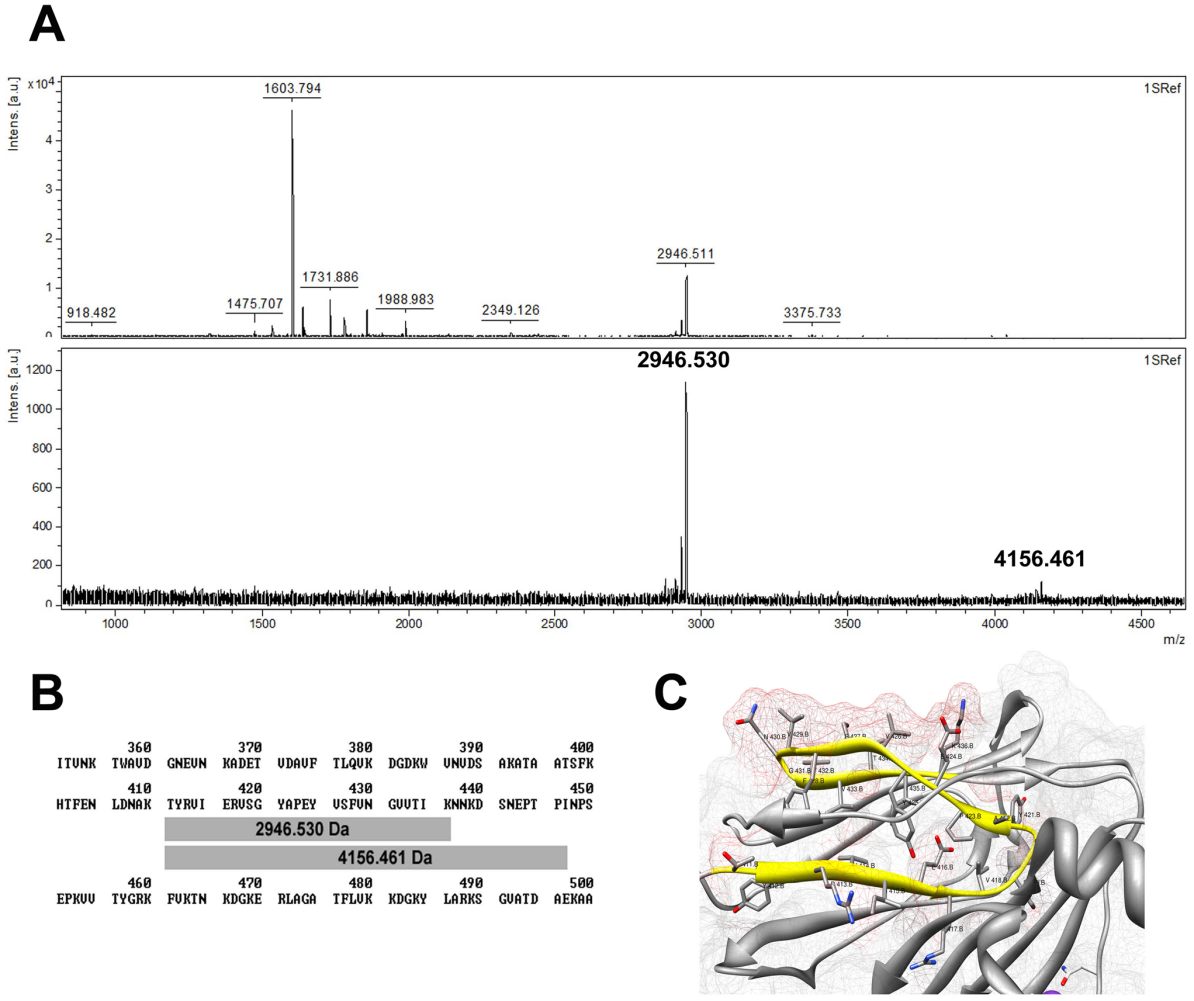
Mapping with functional mAbs identifies an epitope in domain D3. (A) Mass spectrum obtained for the LysC digestion of BP-2a (upper panel) and peptides immunocaptured by mAb 4H11/B7 (lower panel). (B) Sequences corresponding to the immunocaptured peptides (2946.530 Da and 4156.461 Da). (C) Detail of the identified epitope in the structure; in yellow the identified peptide and in red the surface representation of the identified peptide. Note that the segment Glu424 to Lys436 is the most accessible one.

To identify the neutralizing epitope on BP-2a 515 variant, an epitope mapping with the three functionally active monoclonal antibodies (27F2/H2/H9, 17C4/A3 and 4H11/B7) was performed. Two different MS-based approaches were used, one of them allowing the identification of conformational epitopes (see Materials and Methods). With either approach, the experiments were performed six times, using the proteases trypsin, LysC and GluC, and using the mutant form of the entire protein lacking the three isopeptide bonds (BP-2a-515_K199A/K355A/K463A_) previously generated [14]. It is well-known that the presence of internal isopeptide bonds is important for the stability and resistance to proteolysis of single structurally independent domains in which the protein is organized. The results indicate that the three monoclonal antibodies recognize the same region of BP-2a-515 in domain D3, with sequence 411-TYRVIERVSGYAPEYVSFVNGVVTIK-436. The domain D3 was the same protein portion previously characterized as the domain carrying most of the epitopes inducing protective antibody responses [14]. **Figure 2A** shows the mass spectrum of the total LysC digestion of BP-2a-515 (upper panel) and that of the peptides immunocaptured with 4H11/B7 (lower panel). The two labeled signals in the lower panel correspond to the fragments of BP-2a-515 sharing the 411–436 sequence (**Figure 2B**). To confirm the sequence of these peptides, MS/MS spectra were obtained for the peak with a m/z = 2946.530 Da. For the peak with a m/z = 4156.461Da no MS/MS was recorded due to the low intensity of the signal. When using trypsin and GluC no immunocaptured peptide fraction could be detected. Since the peptides retained by the antibodies after LysC digestion contained R and E residues, potential cleavage sites for trypsin and GluC, respectively, these results suggest that the residues R and E and their immediate neighbors may play a role either in the interaction with mAbs or in the structural arrangement of the epitope, since cleavage at either site prevents binding. In the BP-2a-515 structure (PDB code: 2XTL) [14] it can be observed that the residues that are exposed at the surface are comprised between Glu424 and Lys 436 (**Figure 2C**). The fact that the two approaches tested (see Materials and Methods) lead to the same result indicates that epitope recognition is primarily based on sequence.

### Functional mAbs bind BP-2a 515 on the same region of D3 domain, centered on the same key residues: VAL429 and ASN430

**Figure 3:**
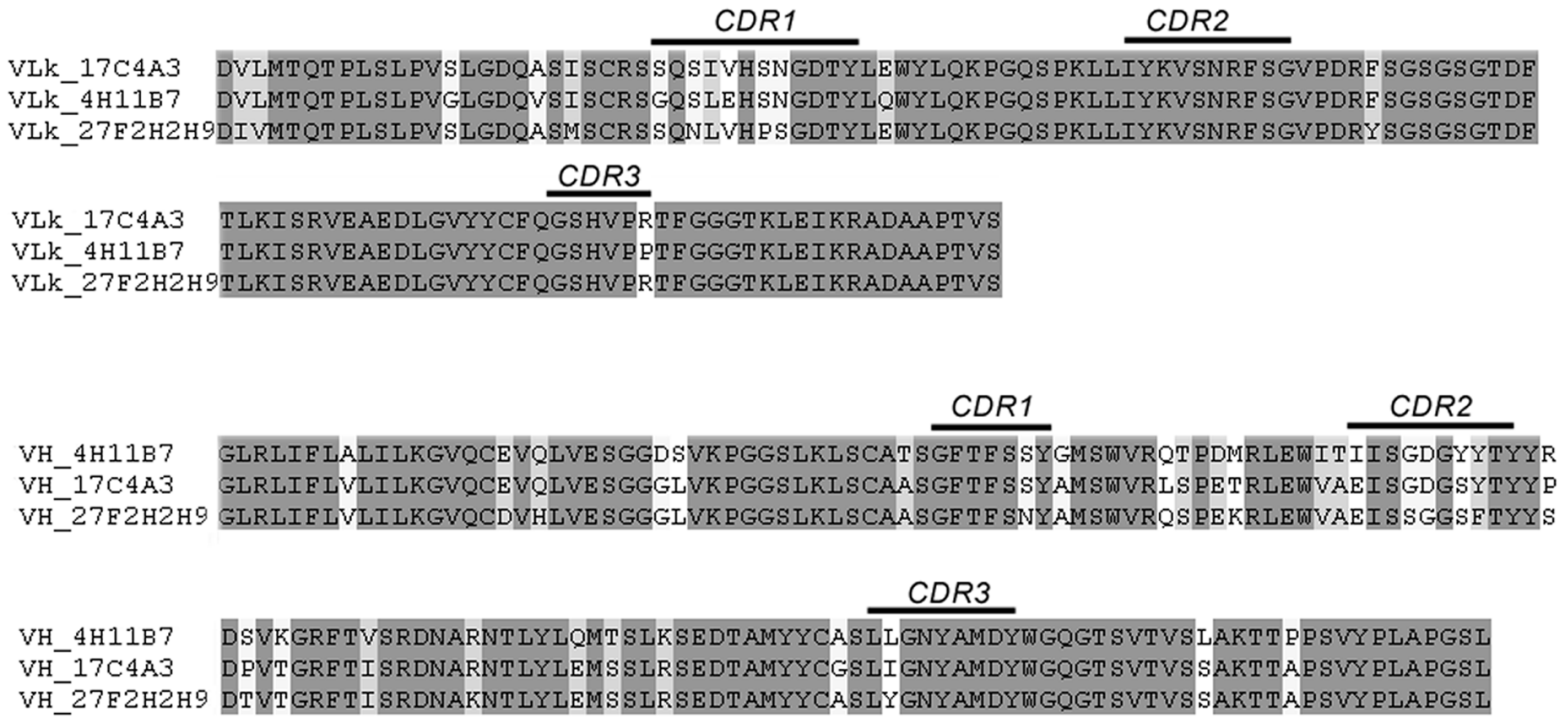
Protein sequences of the three functionally active mAbs. Sequence comparison between the functionally active mAbs selected against BP-2a protein 515 variant. CDR1, CDR2, and CDR3 as defined by Kabat *et al.* [39] are highlighted.

To investigate the mode of action of neutralizing mAbs on the antigen BP-2a 515, we performed mAbs-protein docking. Monoclonal antibodies sequences were obtained by isolation of total RNA from each hybridoma cell line and reverse-transcription. Then, using the generated cDNA as template, the heavy (V_H_) and light (V_L_) chains were amplified using specific PCR primers. Sequence comparison of the three neutralizing mAbs is showed in **Figure 3** and all light chains were of the κ-type.

To elucidate the residue-specific interaction between antigen and antibody at the binding interface, after mAbs sequencing, a structural model of the Fv domain of two of them (17C4/A3 and 4H11/B7) was developed using Modeler 9v8 [15]. Template crystal structures for mAb 17C4/A3 were selected from PDB showing 80% sequence identity for an antibody variable heavy chain (PDB entry 1H3P) and 79% sequence identity for an antibody variable light chain (PDB entry 2ROW). The same procedure led to the selection of two template crystal structures for mAb 4H11/B7 sharing 90% sequence identity for the light chain (PDB entry 1I9J) and 76% sequence identity for the heavy chain (PDB entry 3O6M). In both cases, light and heavy chains were packed together and energy minimized before proceeding with docking studies.

To investigate at the amino-acid level the molecular interactions between neutralizing mAbs and BP-2a antigen, the modeled structures of antibodies were docked against the partial crystal structure of the antigen [14] using ATTRACT [16]. Knowing that mAbs bind to the D3 domain of the antigen in the region T411-K436, we used this information to screen the most accurate complexes within a range of 15000–20000 complexes generated by the docking program.

To validate the stability and reliability of the best selected complexes, we performed explicit solvent molecular dynamics simulation using GROMACS 4.0.5 simulation package [17].

**Figure 4:**
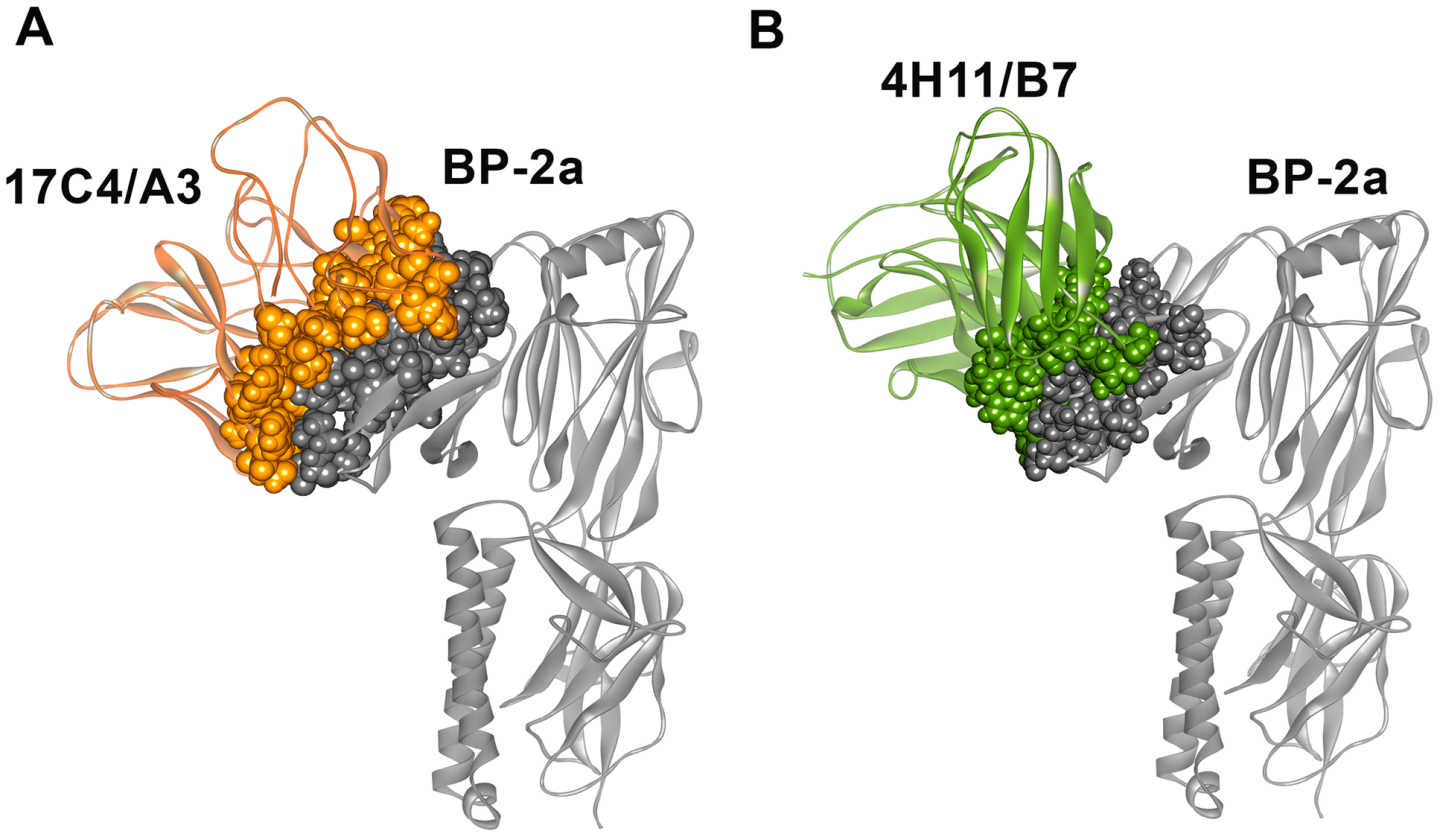
Molecular mAbs/BP-2a 515 variant docking reveals two different binding orientations. (A) Ribbon representation of mAb 17C4/A3-BP-2a 515 variant complex. (B) Ribbon representation of mAb 4H11/B7-BP-2a 515 variant complex. In both complexes, the binding interface is CPK represented and colored according to the mAbs and antigen overall structure.

**Figure 5:**
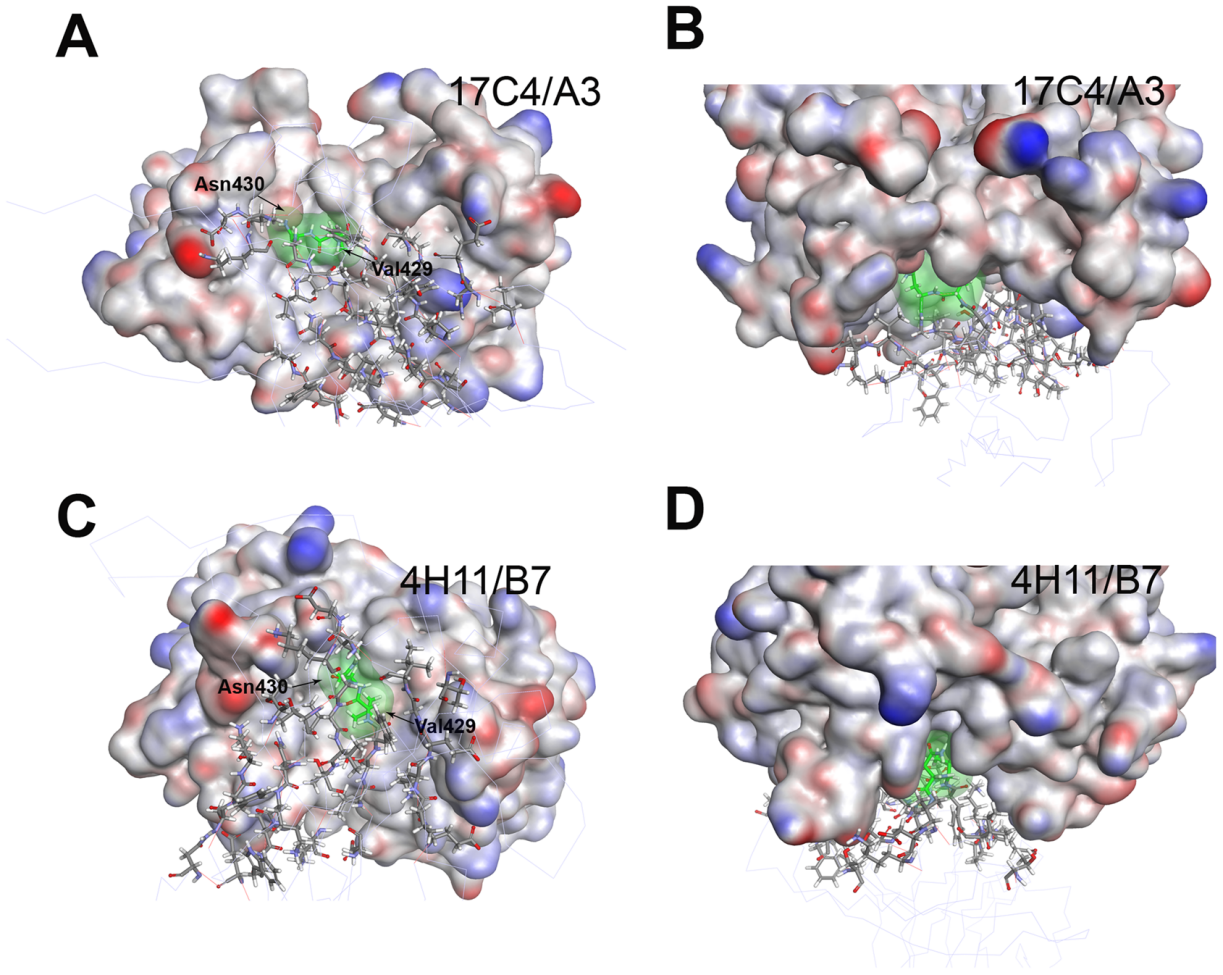
Identification of surface residues that mediate the interaction. (A) Side and (B) front view of mAb 17C4/binding site. (C) Side and (D) front view of mAb 4H11/B7 binding site. In all the panels, the model structure of mAbs is represented as surface, colored by interpolated charge. MAbs are shown in complex with the identified immunogenic peptide (stick) located into the D3 domain of the protein. The residues of the protein antigen, Val429 and Asn430, are represented as green stick and colored surface.

**Figure 6:**
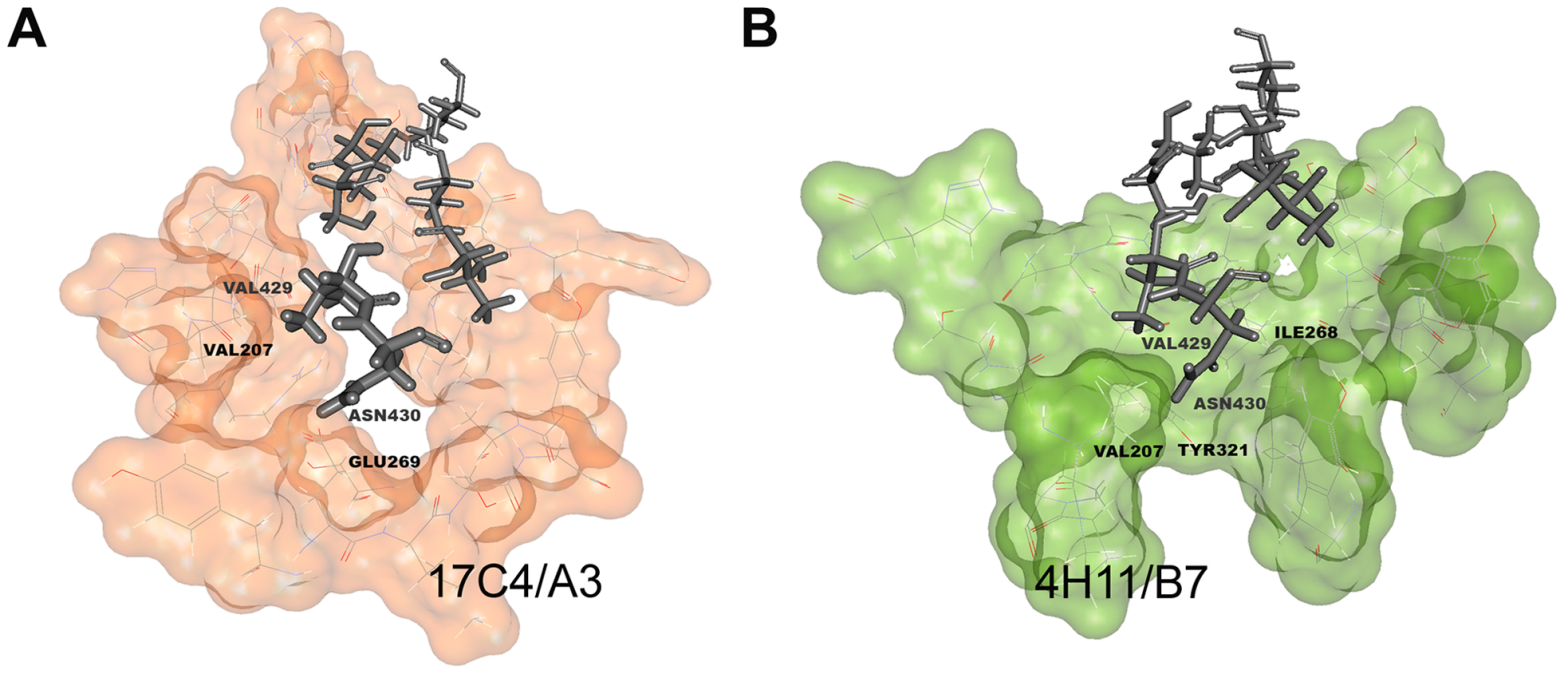
Surface representation of the antibodies binding interface. (A) Deep view of 17C4/A3-peptide complex. Antibody interface is orange colored and the peptide is represented as dark grey stick. (B) Deep view of 4H11/B7-peptide complex. Antibody interface is green colored and the peptide is represented as dark grey stick. In both panels, the fundamental residues for binding interaction are indicated.

Molecular dynamics results of best docked solutions of mAbs/BP-2a antigen revealed different binding orientations of the neutralizing mAbs against the target protein which showed the importance of specific residues both in the epitope and in the paratope. Molecular simulation results indicated that a shorter portion of the previous identified epitope might be necessary at binding interface: P_423_-K_436_ in 17C4/A3-complex (**Figure 4A**) and V_426_-K_436_ in 4H11/B7-complex (**Figure 4B**). In particular, during the course of the simulation, the distance between those residues and the CDR remained at a contact distance of around 4 Angstrom, indicating their importance for complex interaction and stability. Although two different mAb binding orientations were identified as sterically possible, two amino acid residues located on the target antigen were identified as fundamental at the binding interface in both cases: Val429 and Asn430. Remarkably, residue 429 had been identified as being under selective pressure, which is consistent with a direct role in the interaction with the antibody. Both mAb binding interfaces form a deep cleft filled by a loop region of domain D3. In the docking models residues Val429 and Asn430 fill the deeper cavity of both clefts in a water-free environment (**Figure 5**). Val429 is involved in hydrophobic interaction with Val206, Arg208 and Ser204 in the 17C4A3-antigen complex (**Figure 6A**), whereas it interacts with Val207, Leu317, Asn320 and Tyr321 in the 4H11/B7-antigen complex (**Figure 6B**). Asn430 is predicted to interact with residue Glu269 and Arg208 through H-bonds and makes polar interactions with Ala252, Ser254 and Ile318 in the 17C4A3-complex (**Figure 6A**), while it establishes through polar interactions with Tyr321 and Tyr277 residue in the 4H11/B7-complex (**Figure 6B**).

### The epitope is subject to sequence variation and selective pressure

**Figure 7:**
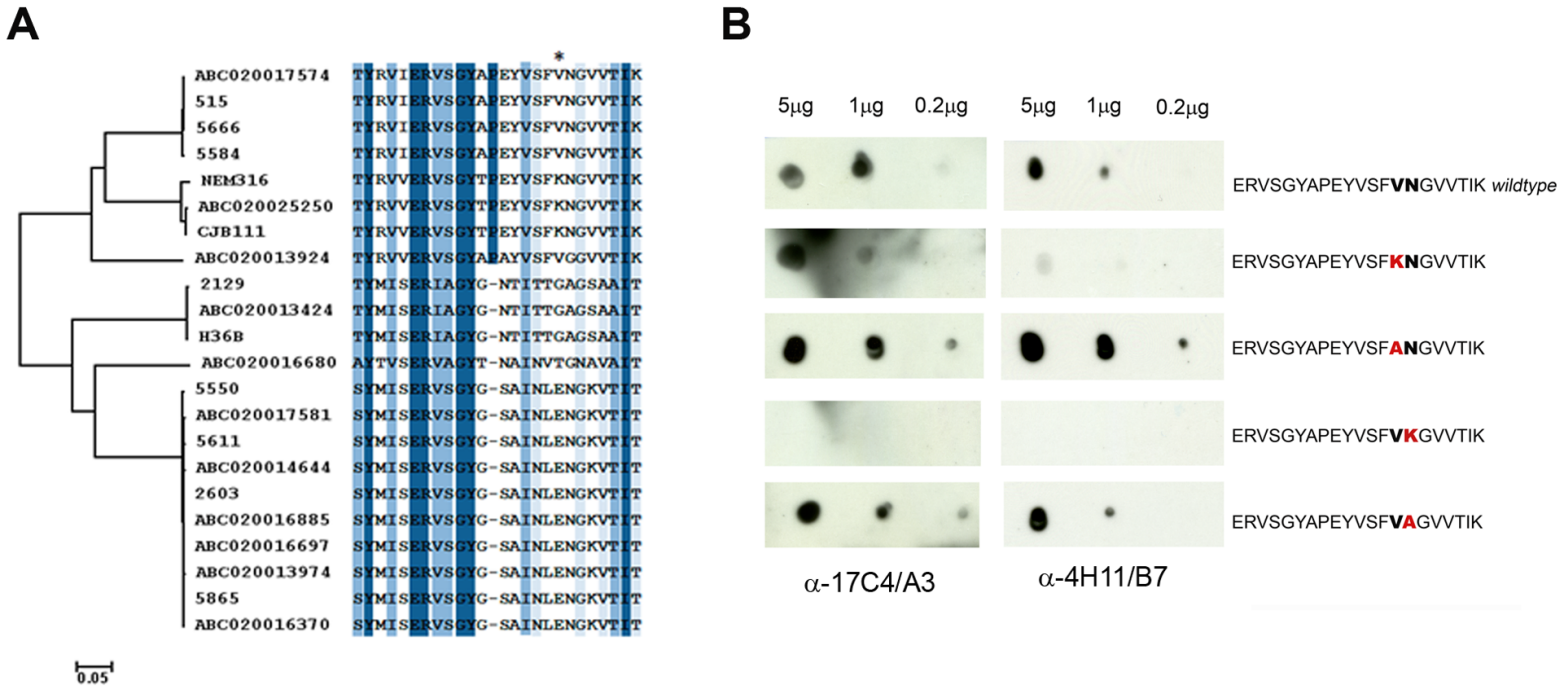
Single amino acid contribution to mAbs-antigen interaction. (A) Sequence alignment of the identified epitope region in the 22 BP-2a allelic variants. The alignment is colored according to sequence identity from dark blue to white. The neighbor-joining phylogenetic tree has been constructed with the full 22 BP-2a protein sequences. The epitope alignment clearly illustrates a mosaic structure in the protein indicating the presence of recombination events. An asterisk indicates a position under positive selection. (B) Peptide dot blot immune assay of wild type and mutated peptide with 17C4/A3 and 4H11/B7 mAbs. The mutated residues within each synthetized peptide are highlighted in red.

The level of conservation of the epitope was analyzed in a set of 144 BP-2a sequences from different GBS isolates. An alignment of the protein sequences revealed six main variants, as described in previous work [13]. In the full protein alignment, inter-variant variability is high (*p* distance = 0.31) with relatively few conserved positions (25%), while intra-variant variability is low, suggesting a mosaic structure (see full alignment in Supplementary Material). Comparative sequence analysis of the identified epitope region among the BP-2a protein sequences divided the isolates into the same variants (**Figure 7A**) with the same variability pattern of the full protein (*p* distance = 0.37 and 26% conserved sites). The alignment also showed that the epitope region has a more conserved first half, residues 411 to 423 (sequence 515), and a more divergent second half, residues 424 to 436 (**Figure 7B**). In addition, the unique BP-2a nucleotide sequences were aligned to study the genetic events causing the observed variability. A recombination analysis using GARD [18] identified two statistically significant break points located at codon positions 198 and 636 (sequence 515). To distinguish the effect of mosaicism and point mutations in epitope variability, results from the GARD algorithm were taken into account for the estimation of positive selection. Thus, the REL algorithm implemented in HyPhy [19] identified 16 sites under selective pressure in BP-2a (Supplementary Material), one of them (Val429, sequence 515) located in the epitope region (**Figure 7A**). These results suggest that both recombination and selection of advantageous mutations have acted to generate the six BP-2a variants for both the full protein and the epitope region. In particular, residue 429 could be changing in response to the pressure of the immune system and be thus a key element for immunological specificity.

To further confirm single amino acid contributions to mAb-epitope binding and elucidate molecular determinants of the immunological specificity of each BP-2a allelic variant, a peptide dot blot immunoassay was performed. Combining epitope mapping, docking and molecular dynamics results with alignment data and detected signatures of positive selection (**Figure 7A**), four mutated peptides were synthesized (**Figure 7B**) and used to evaluate the contribution of single point mutations at the mAb-antigen binding interface. In particular, major consideration was reserved on those residues found to reach the deeper cavity of the mAbs cleft: Val429 and Asn430. The designed mutations were aimed at testing the effect of size and charge of the side chains of these two residues on the binding to the antibodies. Mutating both the Asn430 and Val429 to an alanine residue resulted in a conserved capability of binding to the two mAbs, even with increased affinity in the case of Val429 substitution. On the other hand, mutating either residue to lysine, that carries a bulkier and positively charged side chain, substantially reduced or completely abolished the binding of the mAbs to the spotted peptides (**Figure 7B**). The results from lysine substitution support the presence of residues 429 and 430 at the interaction interface (highly perturbed by a lysine) while those from alanine substitution suggest that these two residues are not responsible for specificity but their nature is probably limited by their fit in the cavity.

## Discussion

The ability of the host to identify microbial molecular determinants that are unique to pathogens has a crucial role in host defense. The recognition by the immune system of the host of surface exposed components, such as proteins and polysaccharides represents the start signal for microbial clearance. Characterization studies of vaccine formulations require a deep knowledge of the interactions between pathogen and host immune system and vaccine components should include the molecular determinants able to stimulate an effective immune response against a specific pathogen. The data reported in this work show a significant correlation between the molecular interactions of monoclonal antibody/target protein with successful neutralizing response against bacterial infection.

The protection against GBS has been associated with the production of high levels of neutralizing antibodies which specifically recognize the antigens exposed on bacterial surface [20], [21]. However, the specific mechanisms, in terms of single molecular determinants, by which antibodies neutralize GBS infections, are not completely understood. Moreover, it is well-known that GBS as well as many other bacteria have evolved a wide range of mechanisms to escape the immune system of their hosts or to adapt to environmental variation, for instance, adopting the strategy of gene variability and/or differential gene expression. These strategies play a crucial role in the capacity of pathogens to trigger disease and also explain why it is so difficult to develop vaccines against these microorganisms. Thus, positive selection and recombination have played an important role in adaptation of the core-genome of different *Streptococcus* species to different hosts [22]. In this context, the identification of different allelic variants of a key vaccine candidate, such as the pilin protein BP-2a, able to induce variant-specific protection [13] clearly reflects a typical strategy of the bacterium to escape the immune system of the host and, at the same time, represent an additional confirmation of the important role of this protein in GBS virulence. Recent data showed that the majority of protective epitopes of the different BP-2a alleles are located in a single structurally independent domain, called D3 domain [14]. A synthetic chimeric protein constituted by the protective D3 domain of the six BP-2a variants was able to protect mice against the challenge with all of the type 2a pilus-carrying strains [14]. In this work we have investigated the contribution of single amino acid residues within the immunodominant domain D3 of BP-2a, able to drive the neutralizing host humoral response. As a tool to investigate the principles governing functional antibody/antigen interactions at the amino acid level, we successfully selected functionally active monoclonal antibodies targeting the 515 allelic variant of the pilin BP-2a. The selected mAbs were able both to recognize the polymerized pilus structure on bacterial surface and to mediate complement-dependent opsonophagocitic killing of live bacteria.

Epitope mapping analysis of two of the neutralizing mAbs identified showed that the mAbs bind to the same region of BP-2a-515 in domain D3, the same domain previously identified as the immunodominat protein region carrying protective epitopes [14]. Although these results confirmed the importance of D3 domain for immunogenicity and protection capacity of BP-2a, to elucidate the specific affinity of the antibodies versus their protein target a structural analysis of the mAbs alone and in complex with the target antigen has been performed. Molecular docking and MD simulation studies indicated that only two specific residues on the target protein, Val429 and Asn430, were able to reach the deepest cavity formed by the antibody binding interface, mediating specific hydrophobic and polar/H-bond interactions, respectively.

Previous studies support the importance of single amino-acid residues at the binding interface in mAb/antigen interactions, responding to a strict balance of shape and energetics. In the case of Influenza virus (H_3_N_2_ vaccine strains 1968–2007), modeling and antigen/antibody docking analyses revealed the molecular basis of the interactions between Hemagglutinin (HA) protein, the primary target of the human immune system, and monoclonal antibodies [6]. Specific mutations both in the neutralizing epitopes and in their vicinity altered the protein surface and the surface electrostatics of the virus, leading to the loss of recognition by the antibody [6]. Though the epitopes responsible for immunity were very similar in successive variants of HA, the simulations could explain the antigenic drift of pathogen surface determinants that has been responsible for the loss of immunity against Influenza infection even in vaccinated population [6]. It has been also shown that single-residue mutants of an antigen may prevent docking by increasing the free energy barrier to conformational rearrangements required for binding to the antibody [23]. In light of this data, the variability of the epitope region identified in BP-2a has been analysed, including the detection of events of recombination and positive selection. Both factors have been found to be significant players in epitope variability. Thus, recombination is likely to be at the basis of the six allelic variants known today and, in addition, residue 429 is predicted to be under positive selection. Interestingly, this residue is located in a part of the epitope region that has low conservation, strengthening the hypothesis of a GBS antigenic drift to escape the immune response and to adapt to the host.

To further characterize the epitope recognized by 4H11/B7 and 17C4/A3 mAbs, a functional dot blot assay using mutated peptides was performed. To identify functional residues within the neutralizing epitope, we mutated those residues predicted to be fundamental at the binding interface (Val429 and Asn430) to lysine and alanine. Mutating Val429 and Asn430 into alanine did not drastically affect mAb surface electrostatics and did not generate steric interferences that could inhibit the binding of antibody. This indicates that a perfect surface antigen-antibody complementarity in this region is not necessary for binding. Conversely, changing the same residues in lysine resulted in a decreased, in the case of Val429, and in a complete abolishment, in the case of Ans430, of mAb binding. The substitution of valine or asparagine by lysine introduces a drastic perturbation of the shape and electrostatics of the antigen surface at this region. The fact that this perturbation inhibits binding supports the presence of these two residues at the interface.

Overall, this study provides new insights into mAbs-BP-2a 515 variant interactions and highlights the molecular correlation between BP-2a variability and its immunological specificity. Moreover, the identification of a neutralizing epitope of a highly immunogenic antigen could be useful for a knowledge-based design of effective vaccines, avoiding the side effects of unfavorable epitope(s) and stringently targeting the immune response only on those one(s), belonging either to the same or to different antigenic protein(s), responsible of pathogenic clearance. Knowing the native molecular architecture of protective determinants could be possible to selectively engineer the antigens for including them in a more effective vaccine formulation.

## Materials and Methods

### Ethics statement

Animal treatments were performed in compliance with the Italian laws and approved by the institutional review board (Animal Ethical Committee) of Novartis Vaccines and Diagnostics, Siena, Italy.

### Bacterial strains, media, and growth conditions

GBS strains used in this work are 515 (serotype Ia, expressing BP-2a-515 allele); CJB111 (serotype V, expressing BP-2a-CJB111); H36B (serotype II, expressing BP-2a-H36B); 3050 (type II, expressing BP-2a-2603); CDC84 (serotype II, expressing BP-2a-DK21); and strain CDC89 (serotype Ia, expressing BP-2a-CJB110). Bacteria were grown at 37°C in Todd Hewitt Broth (Difco Laboratories) or in trypticase soy agar supplemented with 5% sheep blood.

### Cloning and protein purification

The full-length BP-2a 515 variant and the mutated form of BP-2a-515 (BP-2a-515_K199A/K355A/K463A_) were produced as previously reported [14]. Recombinant proteins were expressed in *E. coli* BL21 (DE3) (Novagen) cells as His-tagged fusion proteins and purified by affinity chromatography and gel filtration.

### Generation of mouse monoclonal antibodies

Mouse monoclonal antibodies (mAbs) were generated by Areta *International* (Varese, Italy) using standard protocols. Briefly, B-cell hybridoma clones were isolated from spleen cells of immunized CD1 mice with the purified recombinant BP-2a-515 protein. Positive clones were first selected by ELISA and then culture supernatants were screened for binding to the surface of GBS 515 strain by flow cytometry. Positive primary hybridoma clones were subjected to single cell cloning and sub-cloning by limiting dilution. Monoclonality of a clone was accepted only when all the wells of a microtitre plate with growing cells gave positive reaction in indirect ELISA after repeated sub-cloning. The selected mAbs were finally purified by protein G affinity chromatography. Classes and subclasses of the monoclonal antibodies were determined by IsoQuick Mouse Monoclonal Isotyping Kit (Sigma).

### Flow cytometry analysis

Flow Cytometry Analysis (FACS) analysis was performed as described elsewhere [9]. Briefly, mid-exponential phase bacterial cells were fixed in 0.08% (wt/vol) paraformaldehyde and incubated for 1 hour at 37°C. Fixed bacteria were then washed once with PBS, resuspended in Newborn Calf Serum (Sigma) and incubated for 20 min. at 25°C. The cells were then incubated for 1 hour at 4°C in presence of mAbs diluted 1∶200 in dilution buffer (PBS, 20% Newborn Calf Serum, 0.1% BSA). Cells were washed in PBS-0.1% BSA and incubated for a further 1 h at 4°C with a 1∶100 dilution of R-Phycoerythrin conjugated F(ab)2 goat anti-mouse IgG (Jackson ImmunoResearch Laboratories; Inc.). After washing, cells were resuspended in PBS and analyzed with a FACS CANTO II apparatus (Becton Dickinson, Franklin Lakes, NJ) using FlowJo Software (Tree Star, Ashland, OR).

### Opsonophagocytosis assay

The assay was performed using differentiated HL-60 as phagocytic cells and live bacteria as target cells. GBS strain 515 was grown to mid-exponential growth phase (A_650 nm_ = 0.3), harvested by centrifugation, and, after washing in cold saline solution, was resuspended in HBSS buffer (Invitrogen). Promyelocytic HL-60 cells (ATCC, CCL-240) were expanded in RPMI 1640 (Invitrogen) containing 10% Fetal clone I (HyClone) at 37°C with 5% CO_2_ and differentiated into granulocyte-like cells to a density of 4×10^5^ cells/ml by the addition of 100 mM N,N dimethylformamide (DMF, Sigma) to the growth medium. After 4 days, cells were harvested by centrifugation and resuspended in HBSS buffer. Briefly, the reactions took place in a total volume of 125 µl containing ≈3×10^6^ differentiated HL-60, ≈1,5×10^5^ CFU of GBS cells, 10% baby rabbit complement (Cedarlane), and different dilutions of purified mAbs. Immediately before and after 1 h of incubation at 37°C with shaking at 350 rpm, a 25-µl aliquot was diluted in sterile distilled water and plated onto trypticase soy agar plates with 5% sheep blood. A set of negative controls consisted of reactions without phagocytic cells or with heat-inactivated complement. The amount of opsonophagocytic killing (log kill) was determined by subtracting the log of the number of colonies surviving the 1 h assay from the log of the number of CFU at the zero time point.

### Biosensor analyses

Surface plasmon resonance (SPR) analyses were performed using a Biacore X100 instrument (GE Healthcare). Protein A and Protein G (Sigma) were immobilized on CM5 biosensors (Biacore) using standard primary amine coupling (Amine Coupling Kit, GE Healthcare) in which the carboxymethylated CM5 dextran layers were activated by mixing equal volumes of 0.4 M N-ethyl-N′-(3-dimethylaminopropyl)carbodiimide (EDC) and 0.1 M N-hydroxysuccinimide (NHS) at a flow rate of 10 µL/min for 7 min injection. Protein A (250 µg/mL) and Protein G (150 µg/mL) in 10 mM sodium acetate pH 4.5, were immobilized on the activated biosensors using a contact time of 9 min at 10 µL/min flow rate. Unreacted NHS-esters were blocked with three injections (4 min each) of 1.0 M ethanolamine hydrochloride, pH 8.5. The immobilization procedure allowed obtaining a Protein A and a Protein G coated biosensors of ∼3500 RU and ∼1000 RU respectively. Untreated flow cell 1 was used as reference. PBS buffer pH 7.2 with 0.005% (v/v) Tween 20 was used as running buffer for protein immobilization and binding experiments.

To perform single cycle kinetics (SCK), monoclonal antibodies (ranging from 5 to 15 nM in running buffer) were captured onto the Protein A and Protein G surfaces, according to their isotype, at a flow rate of 2 µL/min for 4 min injection.

The analyte BP-2a 515 variant in 2-fold serial dilutions in running buffer (starting from 500 or 250 nM, five concentrations in total) was injected over the captured antibody for 2 min at 45 µL/min followed by a 5 or 10 min dissociation. Biosensor regeneration was performed after each cycle and achieved using urea 8 M, pH 10.5 (4 min, 10 µL/min). This treatment did not damage the biosensor surface as shown by equivalent signals of capturing ligand on different runs. Each kinetic experiment was preceded by an identical binding-regeneration cycle of buffer as analyte after mAb capture. This cycle was used as blank and subtracted from all the active curves to correct background effects.

The association, dissociation and affinity constants (k_a_ e k_d_ and K_D_ respectively) were determined by a simultaneous fitting of the kinetic curves with a model of equimolar stoichiometry (1∶1) using the BIAevaluation X100 software version 1.0 (GE Healthcare).

### Epitope mapping with monoclonal antibodies

The epitope-mapping approach was based on the method described by Peter and Tomer [24], which we adapted to the following two protocols [25], [26]:

#### 1) Immunocapturing of peptides from antigen partial digestion

Peptide mixtures were obtained by digestion of BP-2a with trypsin, GluC and LysC (independently) in 50 mM ammonium bicarbonate buffer in a ratio 10∶1 at 37°C for 3 h. To capture the epitope-containing peptide, a 25 µl suspension of Dyanbeads Pan Mouse IgG (uniform, superparamagnetic polystyrene beads of 4.5 µm diameter coated with monoclonal human antimouse IgG antibodies) was used. The beads were washed twice with PBS using a magnet and resuspended to the initial volume. 1 µg of the probe (murine) mAb was added and incubated for 30 min at room temperature, after which the beads were washed twice with PBS to remove mAb excess. 0.5 µl of Protease Inhibitor Mix (GE Healthcare) were added prior to the addition of the peptide mixture to avoid potential degradation of the antibodies. The sample was incubated for 30 min at room temperature with gentle tilting and rotation. After incubation the beads were washed three times with 1 ml PBS, and the bound peptide was then eluted with 50 µl of 0.2% TFA. The elute fraction was concentrated and washed with C18 ZipTips (Millipore) and eluted in 3 µl of 50% ACN and 0.1% TFA. For MALDI-MS analysis, 1 µl of sample was mixed with the same volume of a solution of alpha-cyano-4-hydroxy-transcinnamic acid matrix (0.3 mg/ml in H_2_O∶ACN∶TFA at 6∶3∶1), spotted onto the MALDI target plate and allowed to air-dry at room temperature. MALDI mass spectra were recorded in the positive ion mode on an UltrafleXtreme MALDI TOF/TOF instrument (Bruker Daltonics). Ion acceleration was set to 25 kV. All mass spectra were externally calibrated using a standard peptide mixture. For MS/MS analysis, the MASCOT search engine (Matrix Science. London, UK) was used with the following parameters: one missed cleavage permission, 20 ppm measurement for MS and 0.3 Da for MS/MS tolerance. Positive identifications were accepted with p values lower than 0.05. In the searches, methionine residues modified to methionine sulfoxide were allowed.

#### 2) Partial digestion of immunocaptured antigens

To allow the capture of conformational epitopes, the order of the steps in the previous protocol was inverted. The intact protein (20 µg) was added to the beads, allowing binding to the immobilized mAbs. The protease was then added to the sample in a ratio 50∶1, with incubation at 37°C for 3 h. After proteolysis, the beads were washed ten times with 1 ml PBS, and the bound peptide was then eluted as previously described. To avoid the analysis of proteolyzed antibody fragments within the elute fraction, negative controls were carried out where PBS was used instead of protein samples.

### Sequencing of monoclonal antibodies

Approximately 5×10^6^ monoclonal antibody-secreting hybridoma cells were collected. Poly(A)+ RNA was isolated using RNeasy Mini Kit according to the manufacturer's instructions (QIAGEN). cDNA was produced via reverse transcription using ∼2 ug of poly(A)+ RNA template and oligo-(dT)12–18 primer using First Strand cDNA Synthesis kit (Novagen). The resulting cDNA was used as a template for PCR amplification using PfuUltra High-Fidelity DNA Polymerase (Stratagene) and degenerated primers specific for FvH and FvL gene fragments (Mouse Ig-Primer Set, Novagen). PCR timing has been set according to the manufacturer's instruction (Mouse Ig-Primer Set, Novagen). Positive PCR products have been purified using a QIAprep Spin Miniprep Kit (QIAGEN) and sequenced.

### Sequence analysis

A total of 144 *S. agalactiae* BP-2a sequences were examined both from GenBank, including previously published sequences [13], and from complete genome sequences. Codon alignments and phylogenies were constructed using the coding region for each of the 22 unique BP-2a gene sequences with MEGA5 [27]. To detect codons that show signs of adaptive evolution the program HyPhy [19] was used, as implemented in Datamonkey [28]. The codon-based maximum likelihood method REL (Random Effects Likelihood) [29] was used to estimate the dN/dS ratio at every codon in the alignment. The REL method can also take recombination into account, provided that prior to the selection analysis a screening of the sequences for recombination breakpoints is performed. The recombination analysis was performed with GARD [18], using the HKY85 substitution model.

### Comparative homology modeling and molecular docking of mAbs

The corresponding amino acid sequences of monoclonal antibodies were used to search the Protein Data Bank (PDB) in order to retrieve suitable templates for modeling. Ten models for each antibody have been obtained with Moleder 9v8 [30]. The best models were selected according to the objective function scoring. The quality of the refined structures obtained was checked with verify Profile-3D module of Discovery Studio 3.0 (Accelrys).

Molecular docking has been performed with ATTRACT docking program [31]. The docking protocol of ATTRACT has already been described in previous publications [16], [31]. Briefly, the antibodies and the target protein (BP-2a 515 variant) coordinates are translated into a reduced protein presentation made up to three pseudo atoms per amino acid residue: the protein backbone is represented by one pseudo atom, small aminoacid side chains (Ala, Asp, Asn, Cys, Ile, Leu, Pro, Ser, Thr, Val) are represented by one pseudo atom and larger and more flexible side chains are represented by two pseudo atoms, to better describe the shape and dual chemical character of side chains. The contacts between pseudo atoms are described by different interaction: Lennard–Jones (LJ)-type potentials (A/r^8^-B/r^6^-potential), repulsive and attractive LJ-parameters describing approximately the size and physico-chemical character of the side chain chemical groups [31]. For systematic docking studies, the antibody, called the ligand protein, was used as probe and placed at various positions and various orientations on the surface of the domain D3 of BP-2a 515 crystal structure. We also took into account the experimental data from Mass analysis, setting a weight of 1.5 on exposed aminoacid residues identified and on the surface area (4A) around them. Best docked complexes were selected according to energy scoring function and were finally energy-minimized using the Sander program from the Amber8 package.18. During energy minimization, a Generalized Born (GB) model was employed to implicitly account for solvation effects as implemented in Amber8.

### Molecular simulations

All molecular dynamics (MD) simulations were performed with the GROMACS 4.0.5 simulation package [17] using the AMBER99SB-ILDN force field [32] with explicit water (TIP3P) [33]. The selected energy minimized complexes served as starting structure for MD simulations. After stepwise heating of the systems to 310 K production runs were performed for up to 20 ns with a time step of 2 fs in the NPT ensemble at 310 K and 1 bar. Temperature and pressure were controlled by Nosé-Hoover [34], [35] (coupling constant tt  = 2.5) and Parrinello-Rahman [36], [37] (tp = 5.0 ps) schemes, respectively. Figures of the molecular structures were generated with VMD [38] and Discovery Studio 3.0 (Accelrys).

### Dot blot immunoassay

Amounts of 5 – 2 – 0.2 µg of purified peptides (Thermo scientific) were spotted on nitrocellulose membrane (0.45 µm pore size, Biorad) and left to dry for at least 30 minutes at room temperature. The spotted membranes were washed three times with PBST (0.05% Tween 20 in phosphate-buffered saline or PBS pH 7.4) applying a constant vacuum flow using SNAP i.d. Protein Detection System (Millipore) and blocked for 1 h at room temperature in PBST buffer containing 10% of non-fat-dry milk (Biorad). The membranes were then probed 1 h at room temperature with specific anti-BP2a mAb (diluted ∼4.5 µg/mL in PBST/1% non-fat-dry milk) and washed 5 minutes (3X) with PBST and further incubated in PBST/1% non-fat-dry milk for 1 h containing a dilution of 1∶1000 goat anti-mouse horseradish peroxidase-conjugated secondary antibody (Dako, Glostrup, Denmark). Subsequently, the filters were washed 15 minutes (2X) with PBST and developed by enhanced chemiluminescence (ECL) detection assay (Pierce ECL Western blotting substrate, Thermo Fisher Scientific Inc.) following manufacturer's protocols.

## Supporting Information

Figure S1 **Amino acid sequences of 22 GBS pilus 2a backbone proteins (BP-2a) belonging to unique nucleotide sequences and aligned with CLUSTALW.** Shaded residues are those that do not differ from the consensus sequence (identical in dark gray and similar in light gray). The rest of the residues are colored according to their physico-chemical properties. The neutralizing epitope segment is boxed. The sites detected to evolve under selective pressure with p<0.05 are indicated with asterisks. The predicted recombination breakpoints in the gene are shown at the top of the alignment, where each colored bar represents a proposed recombinant block.
(DOCX)
