# Supplementary material for: Understanding the Molecular Determinants Driving the Immunological Specificity of the Protective Pilus 2a Backbone Protein of Group B Streptococcus
Source: PLoS Comput Biol. 2013 Jun 27;9(6):e1003115. doi: 10.1371/journal.pcbi.1003115 (PMC3694817; doi:10.1371/journal.pcbi.1003115)

**Figure S1**. Amino acid sequences of 22 GBS pilus 2a backbone proteins (BP-2a) belonging to unique nucleotide sequences and aligned with CLUSTALW. Shaded residues are those that do not differ from the consensus sequence (identical in dark gray and similar in light gray). The rest of the residues are colored according to their physico-chemical properties. The neutralizing epitope segment is boxed. The sites detected to evolve under selective pressure with *p*<0.05 are indicated with asterisks. The predicted recombination breakpoints in the gene are shown at the top of the alignment, where each colored bar represents a proposed recombinant block.


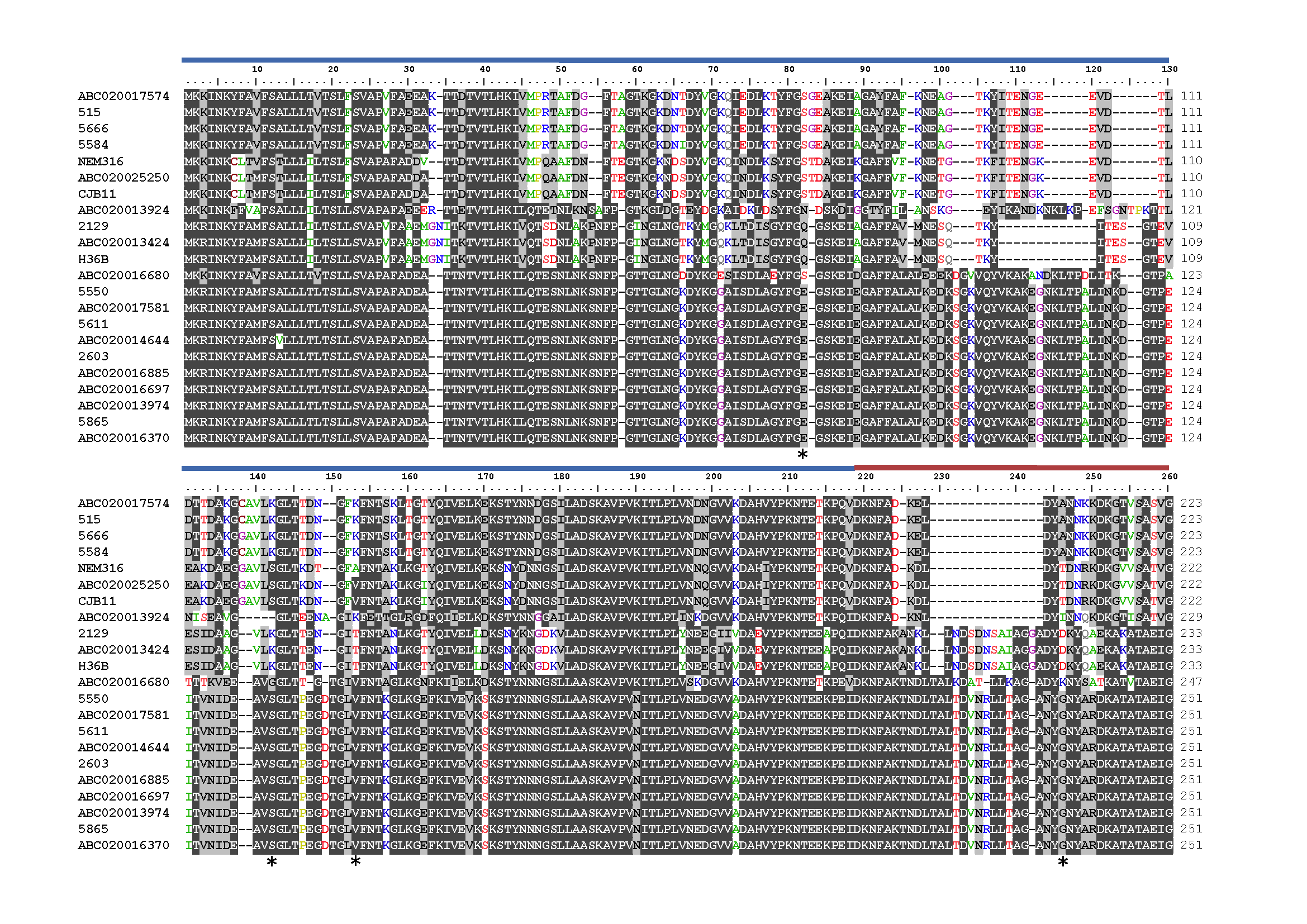


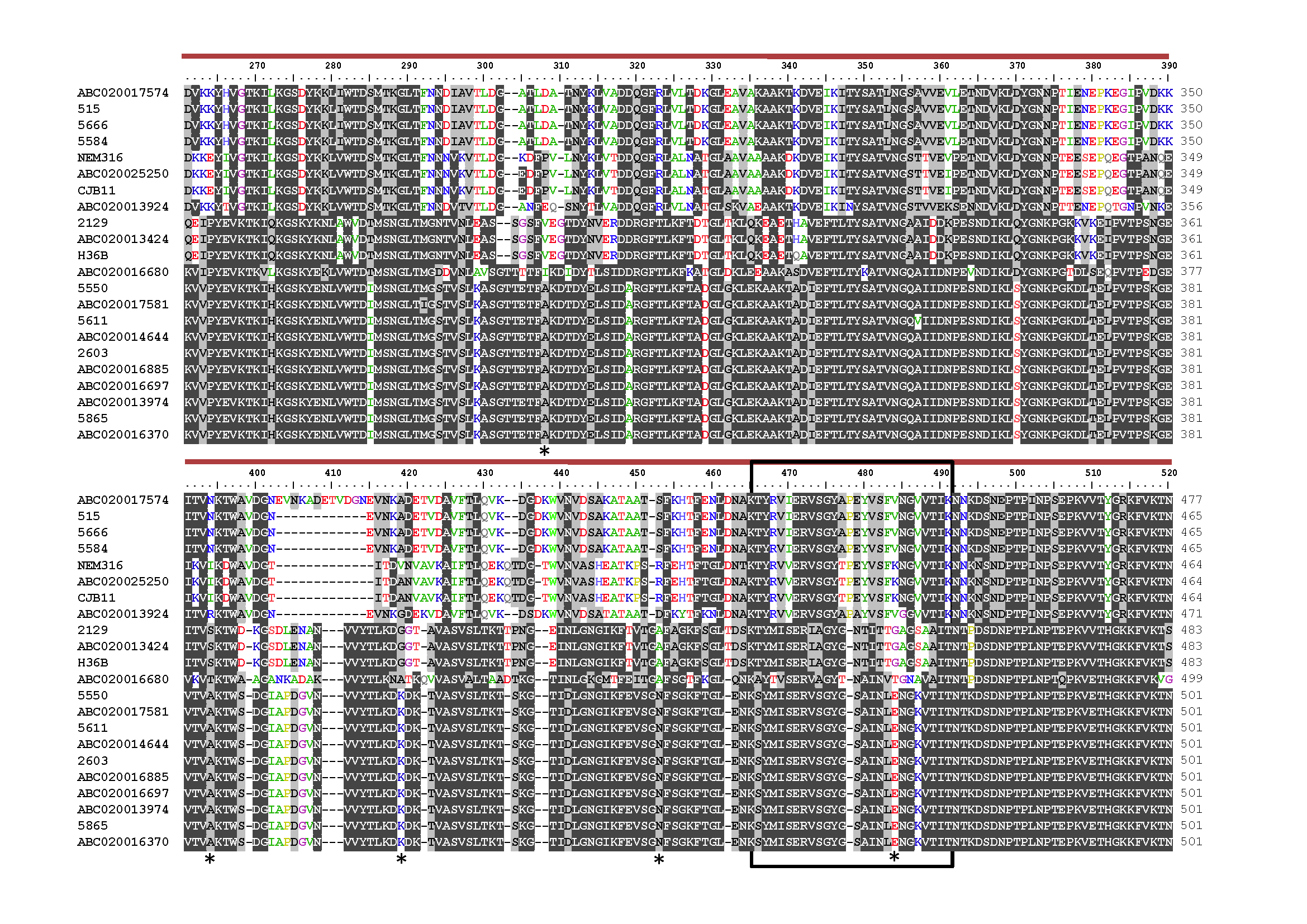


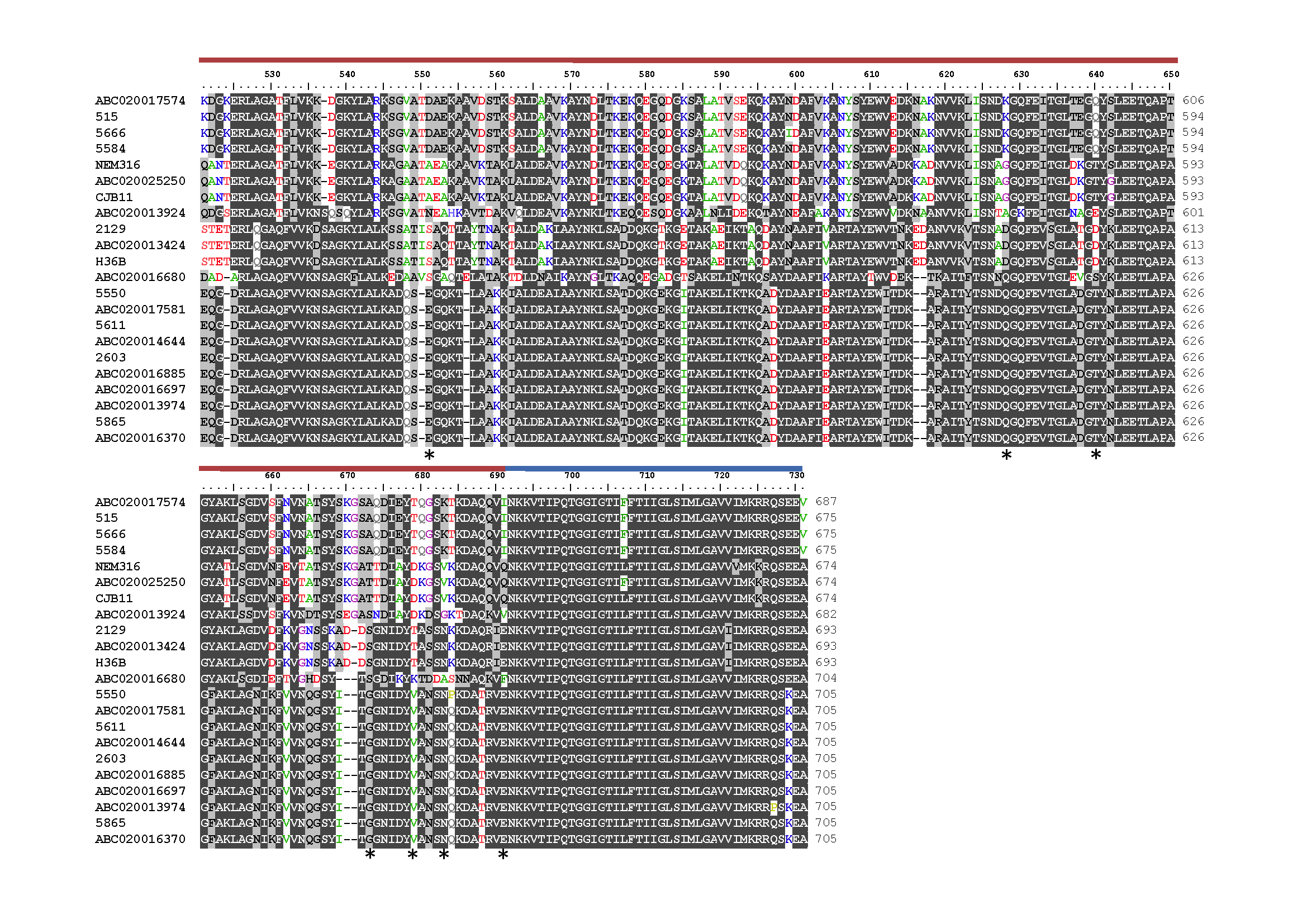

Supplement: Figure S1 — Amino acid sequences of 22 GBS pilus 2a backbone proteins (BP-2a) belonging to unique nucleotide sequences and aligned with CLUSTALW. Shaded residues are those that do not differ from the consensus sequence (identical in dark gray and similar in light gray). The rest of the residues are colored according to their physico-chemical properties. The neutralizing epitope segment is boxed. The sites detected to evolve under selective pressure with p<0.05 are indicated with asterisks. The predicted recombination breakpoints in the gene are shown at the top of the alignment, where each colored bar represents a proposed recombinant block. (DOCX) [file pcbi.1003115.s001.docx]
